# Simultaneous optimized orthogonal matching pursuit with application to ECG compression

**DOI:** 10.1371/journal.pone.0325555

**Published:** 2025-06-11

**Authors:** Laura Rebollo-Neira

**Affiliations:** Department of Applied Mathematics and Data Science, Aston University, Birmingham, United Kingdom; Mae Fah Luang University, THAILAND

## Abstract

A greedy pursuit strategy which finds a common basis for approximating a set of similar signals is proposed. The strategy extends the Optimized Orthogonal Matching Pursuit approach to selecting the subspace containing the approximation of all the signals in the set. The method, called Simultaneous Optimized Orthogonal Matching Pursuit, is stepwise optimal in the sense of minimizing at each iteration the mean square error norm of the signals in the set. When applied to compression of electrocardiograms, significant gains over other transformation based compression techniques are demonstrated on the MIT-BIH Arrhythmia dataset.

## 1 Introduction

Important signals in everyday life such as natural images, audio, and electrocardiogram records, are in general highly compressible. This implies that the original signal, available as a large set of numerical values, can be transformed into a set of much smaller cardinality or a set containing a large proportion of zero values. The transformation, which should not compromise the informational content of the data, is frequently called sparse representation. Traditional methods for sparse representation of signals are realized by applying an orthogonal transformation and disregarding the least relevant points in the transformed domain. Subsequently the signal is recovered by means of the inverse transformation. However, alternative transformations, which are not orthogonal but adapted to a signal at hand, have been shown to render high level of sparsity. Such transformations aim at representing a signal as a superposition of elements, which are called ‘atoms’ and are selected from a large set called ‘dictionary’. The superposition is said to be sparse if it involves a number of atoms much smaller than the number of numerical values representing the original signal.

Given a dictionary, the problem of finding the sparsest approximation of a signal, up to some acceptable error, is an NP-hard problem [1]. In practice it is addressed by tractable methodologies known as Pursuit Strategies. Such methodologies can be grouped for the most part in two broad categories. Namely, Basis Pursuit and Greedy Pursuit Strategies. The Basis Pursuit (BP) approach endeavors to obtain a tractable sparse solution by minimization of the 1-norm [2]. Greedy algorithms seek for a sparse solution by stepwise selection of dictionary’s atoms. When dealing with real data the latter are in general more convenient. From the seminal Matching Pursuit (MP) [3] and Orthogonal Matching Pursuit (OMP) [4] methods, a number of Greedy Pursuit Strategies have been developed to improve the process of sparsely representing single signals [5–16]. Due to complexity issues and memory requirements, most of these techniques are to be applied by segmenting the signal and approximating each segment independently of the others. Nonetheless, when the segments bear similarity with each other, for some applications it is convenient to look for the dictionary’s atoms suitable to represent all the segments simultaneously. The greedy Pursuit Strategy which has been dedicated to simultaneously approximate a set of signal is based on OMP [4] and has been termed Simultaneous Orthogonal Matching Pursuit (SOMP) [17]. Since in this work we extend the Optimized Orthogonal Matching Pursuit method [6] to simultaneously approximate a set of signals, we term the new approach Simultaneous Optimized Orthogonal Matching Pursuit (SOOMP).

The difference between SOMP and the SOOMP approach introduced in this work is equivalent to the difference between OMP and OOMP methods, both for approximating single signals. OOMP is stepwise optimal in the sense of minimizing at each iteration the norm of the residual error. Whilst OMP minimizes the norm of the error only with respect to the coefficients of the atomic superposition, OOMP minimizes the norm of the error with respect to those coefficients *and* the selection of a new atom. In the case of multiple signals SOOMP is designed to minimize the mean value of the error norm squared. An additional advantage arises from the proposed implementation. Based on adaptive biorthogonalization, the SOOMP method produces at each iteration the common dual basis to the basis of selected atoms. This allows to calculate the coefficients of the representation of each signal in the set simply by computation of inner products. We implement the previous SOMP method in an equivalent manner and compare the two approaches for the approximation of stereo music, by selecting atoms from a highly coherent trigonometric dictionary. The practical relevance of the SOOMP approach is further illustrated by using it for compression of electrocardiogram (ECG) records.

An ECG signal represents a sequence of heartbeats which, if properly segmented and aligned, are suitable to be simultaneously approximated. This property is shown to benefit compression. Reliable comparison with other compression techniques is made possible by recuse to an adaptive quantization procedure that facilitates to reconstruct the whole ECG record at the required quality. The compression results are shown to significantly improve upon results produced by different transformation based approaches.

The paper is organized as follows: Sect 2 introduces the problem and the mathematical notation. Sect 3 establishes the proposed SOOMP approach for simultaneous approximation of a set of similar signals. Sect 4 compares the SOOMP and SOMP approaches for the simultaneous approximation of stereo music. Sect 5 applies the proposed SOOMP method for compressing digital ECG records and produces reliable comparisons with previously reported results. The conclusions are presented in Sect 6.

## 2 Mathematical introduction of the problem

In order to pose in mathematical terms the problem to be addressed we need to introduce the notation used throughout the paper as well as some preliminary background.

The sets of real, integer, and natural numbers are indicated by ℝ,ℤ, and ℕ, respectively. Boldface letters are used to indicate Euclidean vectors or matrices whilst standard mathematical fonts indicate components, e.g., $𝐟\in {\mathbb{R}}^{N},\;N\in \mathbb{N}$ is a vector of components f(i),i=1,…,N and $𝐂\in {\mathbb{R}}^{Q\times k}$ is a matrix of elements C(i,j),i=1,…,Q,j=1,…,k which when not leaving room for ambiguity will also be represented as C(:,j),j=1,…,k. A set of *Q* signals of equal length *N*, to be simultaneously approximated in a common subspace, is represented as a set of vectors $\left\{𝐟\left\{q\right\}\in {\mathbb{R}}^{N},\;q=1,\ldots ,Q\right\}$. The inner product is indicated as ⟨·,·⟩, e.g. for $𝐟\{1\}\in {\mathbb{R}}^{N}$ and $𝐟\{2\}\in {\mathbb{R}}^{N}$


$$\left\langle 𝐟\{1\},𝐟\{2\}\right\rangle =\sum_{i=1}^{N}f\{1\}(i)f\{2\}(i).$$

The 2-norm induced by the inner product is denoted as ‖·‖, e.g. for $𝐟\left\{q\right\}\in {\mathbb{R}}^{N}$


$$\|𝐟\left\{q\right\}\|=\sqrt{\left\langle 𝐟\left\{q\right\},𝐟\left\{q\right\}\right\rangle }=\sqrt{\sum_{i=1}^{N}{\left(f\{q\}(i)\right)}^{2}}.$$

A set of *M* vectors


$$𝒟={\left\{{𝐝}_{n}\in {\mathbb{R}}^{N}\;;\|{𝐝}_{n}\|=1\right\}}_{n=1}^{M},$$

such that $\text{span}\left(𝒟\right)={\mathbb{R}}^{N}$ and *N* < *M*, is called a redundant *dictionary* for ${\mathbb{R}}^{N}$ and its elements are called *atoms*.

In our context a signal $𝐟\in {\mathbb{R}}^{N}$ is assumed to be well approximated by an element, say ${𝐟}^{k}$, belonging to a finite dimensional subspace $V_{k}\subset {\mathbb{R}}^{N}$. This assumption implies that, within a tolerance ρ much larger than the numerical errors in the calculations, ${𝐟}^{k}\in V_{k}$ is accepted to be a good approximation of $𝐟\in {\mathbb{R}}^{N}$ if $\|𝐟-{𝐟}^{k}\|<\rho .$ Examples of signals fulfilling this definition are, amongst others, audio signals, and electrocardiograms. These are all signals with acceptable approximations which, without affecting their informational content, do not necessarily produce a highly accurate point-wise reproduction of the signals. These type of signals are suitable for lossy compression.

Since this work concerns approximation of similar signals we need to make an assumption on the signals that will be considered. We say that a finite set of *Q* signals $\{𝐟\left\{q\right\}\in {\mathbb{R}}^{N}{\}}_{q=1}^{Q}$ are similar if they can be well approximated in a subspace $V_{k}$ of dimension *k*, with *k* significantly smaller than *N*. This is equivalent to assuming that there exists a common basis $\{{𝐝}_{\ell_{n}}{\}}_{n=1}^{k}$ for $V_{k}$ such that each signal 𝐟{q} is approximated as


$$\tilde{𝐟}\{q\}=\sum_{n=1}^{k}c\{q\}(n){𝐝}_{\ell_{n}},\;q=1,\ldots ,Q.$$

The quality of the approximated set will be assessed in mean value


$$\overset{―}{ℰ}=\sum_{q=1}^{Q}p(q)\|𝐟\left\{q\right\}-\tilde{𝐟}\{q\}{\|}^{2},$$

where p(q)≥0 with $\sum_{q=1}^{Q}p(q)=1$.

## 3 Strategy for simultaneous approximation of a set of signals

Given a set of similar signals $\{𝐟\left\{q\right\}\in {\mathbb{R}}^{N}{\}}_{q=1}^{Q}$ and a dictionary, the aim is to simultaneously approximate all the signals in the set $\{𝐟\left\{q\right\}\in {\mathbb{R}}^{N}{\}}_{q=1}^{Q}$ within a common subspace $V_{k}=\text{span}\left(\{{𝐝}_{\ell_{n}}{\}}_{n=1}^{k}\right)$. In other words, each signal $𝐟\left\{q\right\}\in {\mathbb{R}}^{N}$ is to be approximated as a *k*-term atomic superposition

$$𝐟{\left\{q\right\}}^{k}=\sum_{n=1}^{k}c\{q\}(n){𝐝}_{\ell_{n}},\;q=1,\ldots ,Q,$$ (1)

where the atoms ${𝐝}_{\ell_{n}},\;n=1,\ldots ,k$ in (1) are selected from the given dictionary according to the criterion of optimality that will be established by Proposition 1 in the next subsection. Let us suppose for the moment that these atoms are known. Assigning a weight p(q)≥0 to the signal 𝐟{q}, with $\sum_{q=1}^{Q}p(q)=1$, the coefficients $𝐜\{q\}\in {\mathbb{R}}^{k}$ in (1) are required to minimize the mean value of the square norm of the errors in the approximation of the set of signals, i.e.

$$𝐜\{q\},\ldots ,𝐜\{Q\}=*{arg\;min}_{{𝐜}^{\prime }\{q\},\ldots ,{𝐜}^{\prime }\{Q\}}\sum_{q=1}^{Q}p(q)\|𝐟\left\{q\right\}-\sum_{n=1}^{k}c^{\prime }\{q\}(n){𝐝}_{\ell_{n}}{\|}^{2}.$$ (2)

Since p(q)≥0 the above minimization is equivalent to finding the components c{q}(n),n=1,…,k of each vector 𝐜{q} such that

$$c\{q\}(1),\ldots ,c\{q\}(n)=*{arg\;min}_{c^{\prime }\{q\}(1),\ldots ,c^{\prime }\{q\}(n)}\|𝐟\left\{q\right\}-\sum_{n=1}^{k}c^{\prime }\{q\}(n){𝐝}_{\ell_{n}}{\|}^{2}\;\;q=1\ldots ,Q.$$ (3)

Accordingly, the minimization with respect to the coefficients in (1) can be implemented by adaptive biorthogonalization [18], as proposed within the OOMP algorithm for a single signal [6],

$$c\{q\}(n)=\left\langle \beta_{n}^{k},𝐟\left\{q\right\}\right\rangle ,\;q=1,\ldots ,Q,$$ (4)

with vectors $\beta_{n}^{k}$ calculated as will be described in the next section.

The selection of the atoms ${𝐝}_{\ell_{n}},\;n=1\ldots ,k$ in the decomposition (1) such that


$$\sum_{q=1}^{Q}p(q)\|𝐟\left\{q\right\}-\sum_{n=1}^{k}c\{q\}(n){𝐝}_{\ell_{n}}{\|}^{2}\;\;\;\text{is minimized}$$

poses an intractable problem (for a dictionary of *M* atoms there are $\frac{M!}{(M-k)!k!}$ possibilities to be checked). We address the selection in a tractable manner by extending the OOMP strategy to simultaneously approximate a set of similar signals. The extended strategy is refereed to as SOOMP (Simultaneous OOMP).

### 3.1. SOOMP algorithm

The algorithm is initialized by setting: $𝐫{\left\{q\right\}}^{0}=𝐟\left\{q\right\}$, $𝐟{\left\{q\right\}}^{0}=0$, Γ=∅ and *k* = 0. The first atom is selected as the one corresponding to the index $\ell_{1}$ such that

$$\ell_{1}=*{arg\;max}_{n=1,\ldots ,M}\sum_{q=1}^{Q}p(q){\left|\left\langle {𝐝}_{n},𝐫{\left\{q\right\}}^{0}\right\rangle \right|}^{2}.$$ (5)

This first atom is used to assign ${𝐰}_{1}=\beta_{1}={𝐝}_{\ell_{1}}$, calculate $𝐫{\left\{q\right\}}^{1}=𝐟\left\{q\right\}-{𝐝}_{\ell_{1}}\left\langle {𝐝}_{\ell_{1}},𝐟\left\{q\right\}\right\rangle$ and iterate as prescribed below.

(1) Upgrade the set $\Gamma \leftarrow \Gamma \cup \ell_{k+1}$, increase k←k+1, and select the index of a new atom for the approximation as
  ℓk+1=*argmaxn=1,…,Mn∉Γ∑q=1Qp(q)|⟨𝐝n,𝐫{q}k⟩|21−∑i=1k|⟨𝐝n,w~i⟩|2,withw~i=𝐰i‖𝐰i‖. (6)
(2) Compute the corresponding new vector ${𝐰}_{k+1}$ as
  $${𝐰}_{k+1}={𝐝}_{\ell_{k+1}}-\sum_{i=1}^{k}\frac{{𝐰}_{i}}{\|{𝐰}_{i}{\|}^{2}}\left\langle {𝐰}_{i},{𝐝}_{\ell_{k+1}}\right\rangle ,$$ (7)
  including for numerical accuracy the re-orthogonalization step:
  $${𝐰}_{k+1}\leftarrow {𝐰}_{k+1}-\sum_{i=1}^{k}\frac{{𝐰}_{i}}{\|{𝐰}_{i}{\|}^{2}}\left\langle {𝐰}_{i},{𝐰}_{k+1}\right\rangle .$$ (8)
(3) Upgrade vectors $\beta_{n}^{k}$ as
  $$\beta_{k+1}^{k+1}=\frac{{𝐰}_{k+1}}{\|{𝐰}_{k+1}{\|}^{2}},\;\beta_{n}^{k+1}=\beta_{n}^{k}-\beta_{k+1}^{k+1}\left\langle {𝐝}_{\ell_{k+1}},\beta_{n}^{k}\right\rangle ,\;n=1,\ldots ,k.$$ (9)
(4) Update $𝐫{\left\{q\right\}}^{k}$ as
  $$𝐫{\left\{q\right\}}^{k+1}=𝐫{\left\{q\right\}}^{k}-\left\langle {𝐰}_{k+1},𝐟\left\{q\right\}\right\rangle \frac{{𝐰}_{k+1}}{\|{𝐰}_{k+1}{\|}^{2}}.$$ (10)
(5) If the stopping criterion is met finish the iterations. Otherwise repeat steps (1)–(5).

**Note**: given a tolerance ρ, as stopping criterion we set:

$$\sum_{q=1}^{Q}p(q)\|𝐫{\left\{q\right\}}^{k+1}{\|}^{2}<\rho$$ (11)

or

$$\sum_{q=1}^{Q}p(q)\|𝐫{\left\{q\right\}}^{k+1}\|<\rho ,$$ (12)

depending on convenience for the particular application.

Once the iterations have finished calculate the coefficients for the decomposition (1) as


$$c\{q\}(n)=\left\langle \beta_{n}^{k},𝐟\left\{q\right\}\right\rangle ,\;n=1,\ldots ,k,\;q=1,\ldots ,Q.$$

For q=1,…,Q calculate the final approximation of each signal 𝐟{q} as


$$𝐟{\left\{q\right\}}^{k}=𝐟\left\{q\right\}-𝐫{\left\{q\right\}}^{k}.$$

**Remark 1.** *The set of vectors $\beta_{n}^{k},\;n=1,\ldots ,k,\;q=1,\ldots ,Q$ as given in (9) fulfills that*


$$𝐟{\left\{q\right\}}^{k}=\sum_{n=1}^{k}\left\langle \beta_{n}^{k},𝐟\left\{q\right\}\right\rangle {𝐝}_{\ell_{n}}={\hat{P}}_{V_{k}}𝐟\left\{q\right\},\;q=1,\ldots ,Q,$$

*where ${\hat{P}}_{V_{k}}𝐟\left\{q\right\}$ is the orthogonal projector of 𝐟{q} onto $V_{k}=\text{span}\{{𝐝}_{\ell_{n}}{\}}_{n=1}^{k}$. Please find the proof in [6], or as a particular case of the more general proof in [18].*

**Proposition 1.** *The recursive selection of the indices $\ell_{1},\ldots ,\ell_{k}$, as proposed in (6), is stepwise optimal. It minimizes, at each iteration, the mean of the square distance between the set of signals 𝐟{q},q=1,…,Q and their corresponding approximations $𝐟{\left\{q\right\}}^{k},\;q=1,\ldots ,Q$.*

*Proof:* For *k* = 0 it is clear that $\ell_{1}$ selected as in (5) minimizes the mean of the square distance $\overset{―}{{ℰ}^{1}}$ as given by


$$\overset{―}{{ℰ}^{1}}=\sum_{q=1}^{Q}p(q)\|𝐟\left\{q\right\}-𝐟{\left\{q\right\}}^{1}{\|}^{2}=\sum_{q=1}^{Q}p(q)(\|𝐟\left\{q\right\}{\|}^{2}-|\left\langle {𝐝}_{\ell_{1}},𝐟\left\{q\right\}\right\rangle {|}^{2}).$$

Let us assume that the indices $\ell_{1},\ldots ,\ell_{k}$ selected as proposed in (6) minimize, in the specified stepwise sense, the mean square distance


$$\overset{―}{{ℰ}^{k}}=\sum_{q=1}^{Q}p(q)\|𝐟\left\{q\right\}-𝐟{\left\{q\right\}}^{k}{\|}^{2}.$$

We shall prove by induction that if the atoms ${𝐝}_{\ell_{1}},\ldots ,{𝐝}_{\ell_{k}}$ are fixed, at iteration *k*  +  1 the atom ${𝐝}_{\ell_{k+1}}$ selected as in (6) minimizes $\overset{―}{{ℰ}^{k+1}}$. The proof stems from the fact that at iteration *k* the approximation $𝐟{\left\{q\right\}}^{k}$ of each signal 𝐟{q} is the orthogonal projection of 𝐟{q} onto the subspace $V_{k}=\text{span}\{{𝐝}_{\ell_{n}}{\}}_{n=1}^{k}$ (c.f. Remark 1).

Consider that $V_{k}$ is augmented by one element, say ${𝐝}_{\ell_{k+1}}\notin V_{k}$, so that $V_{k+1}=V_{k}$ ⊕ ${𝐝}_{\ell_{k+1}}$, where ⊕ indicates direct sum. The orthogonal projection of each signal 𝐟{q},q=1,…,Q onto $V_{k+1}$ can be expressed as


$$𝐟{\left\{q\right\}}^{k+1}={\hat{P}}_{V_{k+1}}𝐟\left\{q\right\}={\hat{P}}_{V_{k}}𝐟\left\{q\right\}+\frac{{𝐰}_{k+1}}{\|{𝐰}_{k+1}{\|}^{2}}\left\langle {𝐰}_{k+1},𝐟\left\{q\right\}\right\rangle \;\text{with}\;{𝐰}_{k+1}={𝐝}_{\ell_{k+1}}-{\hat{P}}_{V_{k}}{𝐝}_{\ell_{k+1}}.$$

Thus


$$\|𝐟\left\{q\right\}-𝐟{\left\{q\right\}}^{k+1}{\|}^{2}=\|𝐟\left\{q\right\}-{\hat{P}}_{V_{k+1}}𝐟\left\{q\right\}{\|}^{2}$$

$$=\|𝐟\left\{q\right\}-{\hat{P}}_{V_{k}}𝐟\left\{q\right\}-\frac{{𝐰}_{k+1}}{\|{𝐰}_{k+1}{\|}^{2}}\left\langle {𝐰}_{k+1},𝐟\left\{q\right\}\right\rangle {\|}^{2}$$

$$=\|𝐟\left\{q\right\}-{\hat{P}}_{V_{k}}𝐟\left\{q\right\}{\|}^{2}-\frac{|\left\langle {𝐰}_{k+1},𝐟\left\{q\right\}\right\rangle {|}^{2}}{\|{𝐰}_{k+1}{\|}^{2}}.$$

Since $\|𝐟\left\{q\right\}-{\hat{P}}_{V_{k}}𝐟\left\{q\right\}{\|}^{2}$ is optimized and fixed at iteration *k*, it is true that at iteration *k* + 1 the index of the atom which minimizes $\overset{―}{{ℰ}^{k+1}}$ fulfils


ℓk+1=*argmaxn=1,…,Mn∉Γ∑q=1Qp(q)|⟨𝐰k+1,𝐟{q}⟩|2‖𝐰k+1‖2

=*argmaxn=1,…,Mn∉Γ∑q=1Qp(q)|⟨𝐝ℓn−P^Vk𝐝ℓn,𝐟{q}⟩|2‖𝐝ℓn−P^Vk𝐝ℓn‖2. (13)

The proof is concluded using the self-adjoint properties of ${\hat{P}}_{V_{k}}$ to write:


$$\left|\left\langle {𝐝}_{\ell_{n}}-{\hat{P}}_{V_{k}}{𝐝}_{\ell_{n}},𝐟\left\{q\right\}\right\rangle \right|=\left|\left\langle {𝐝}_{\ell_{n}},𝐟\left\{q\right\}\right\rangle -\left\langle {\hat{P}}_{V_{k}}{𝐝}_{\ell_{n}},𝐟\left\{q\right\}\right\rangle \right|$$

$$=\left|\left\langle {𝐝}_{\ell_{n}},𝐟\left\{q\right\}-{\hat{P}}_{V_{k}}𝐟\left\{q\right\}\right\rangle \right|$$

$$=\left|\left\langle {𝐝}_{\ell_{n}},𝐫{\left\{q\right\}}^{k}\right\rangle \right|.$$ (14)

Moreover, since all atoms are normalized and the set $\{{\tilde{w}}_{i}{\}}_{i=1}^{k}$ is an orthonormal basis for $V_{k}$ we have


$${\hat{P}}_{V_{k}}{𝐝}_{\ell_{n}}=\sum_{i=1}^{k}{\tilde{w}}_{i}\left\langle {\tilde{w}}_{i},{𝐝}_{\ell_{n}}\right\rangle ,$$

so that


$$\|{𝐝}_{\ell_{n}}-{\hat{P}}_{V_{k}}{𝐝}_{\ell_{n}}{\|}^{2}=1-\|{\hat{P}}_{V_{k}}{𝐝}_{\ell_{n}}{\|}^{2}=1-\sum_{i=1}^{k}|\left\langle {\tilde{w}}_{i},{𝐝}_{\ell_{n}}\right\rangle {|}^{2},$$

which shows the equivalence between (13) and (6). ◻

**Corollary 1.** *The selection criterion (6) guaranties that, if for n=1,…,k the right hand side of (6) is not zero, the selected elements ${𝐝}_{\ell_{n}},\;n\;=\;1,\ldots ,k$ are linearly independent.*

*Proof:* For *k* = 1 the single atom ${𝐝}_{\ell_{1}}$ is linearly independent. Let us assume that the first *k* selected atoms ${𝐝}_{\ell_{n}},\;n=1,\ldots ,k$ are linearly independent and prove that then the newly selected atom ${𝐝}_{\ell_{k+1}}$ is also linearly independent. The proof is achieved by contradiction. Indeed, if ${𝐝}_{\ell_{k+1}}$ is linearly dependent then ${𝐝}_{\ell_{k+1}}=\sum_{n=1}^{k}a_{n}{𝐝}_{\ell_{n}}$, for some scalers $a_{n},\;n=1,\ldots ,k$, so that ${\hat{P}}_{V_{k}}{𝐝}_{\ell_{k+1}}=\sum_{n=1}^{k}a_{n}{\hat{P}}_{V_{k}}{𝐝}_{\ell_{n}}=\sum_{n=1}^{k}a_{n}{𝐝}_{\ell_{n}}={𝐝}_{\ell_{k+1}}$ and from (14) we gather that the right hand side of (6) is zero. This contradiction leads to conclude that the selected elements ${𝐝}_{\ell_{n}},\;n=1,\ldots ,k$ by criterion (6) are linearly independent. ◻

**Remark 2.** *If the dictionary $𝒟={\left\{{𝐝}_{n}\in {\mathbb{R}}^{N}\;;\|{𝐝}_{n}\|=1\right\}}_{n=1}^{M}$ is complete or over-complete, i.e., dim(span(𝒟))=N, then by selecting N atoms the method can reconstruct the exact signals $𝐟\left\{q\right\}\in {\mathbb{R}}^{N},\;q=1,\ldots ,Q.$ Otherwise if dim(span(𝒟))=S<N the selected atoms provide the orthogonal projections ${\hat{P}}_{V_{S}}𝐟\left\{q\right\},\;q=1,\ldots ,Q$, where $V_{S}=\text{span}\left(𝒟\right)$. It should be stressed, though, that with a suitable dictionary all the signals 𝐟{q},q=1,…,Q are expected to be well approximated in a subspace $V_{k}$ with k significantly smaller than N. Otherwise the representation would not qualify to be sparse.*

**Remark 3.** *The complexity of the SOOMP algorithm, at each iteration, is O(NMQ). For equal weights $p(q)=\frac{1}{Q},\;q=1,\ldots ,Q$ the difference between the complexity of the SOOMP selection criterion (13) and the SOMP one [17] is the denominator in the right hand side of (13). This introduces extra computations of complexity O(NM) so that the order of complexity of both algorithms is equivalent. However, as will be illustrated in the next section, due to the reduction in the number of selected atoms, and hence the number of iterations, the approximation may run faster if SOOMP rather than SOMP is used.*

## 4 Numerical example

We illustrate here the algorithm’s implementation by simultaneously approximating stereophonic music. This type of music, commonly called stereo music, is usually produced by using two independent audio channels $𝐟\{1\}\in {\mathbb{R}}^{N}$ and $𝐟\{2\}\in {\mathbb{R}}^{N}$. The example is dedicated to showing the sparsity obtained with the proposed SOOMP method and the precursor SOMP one, when simultaneously approximating both channels using a highly coherent trigonometric dictionary.

Since the signals structure varies with time, approximations with trigonometric dictionaries are carried out on a partition of the signal. In this example the partition consists of disjoint segments, called frames, which are assumed to be all of the same size *L*. The signal representation is realized by independent approximation of each frame.

As shown in [15,16], for music representation the combination of a Redundant Discrete Cosine Dictionary ${𝒟}^{\text{c}}$ and a Redundant Discrete Sine Dictionary ${𝒟}^{\text{s}}$, defined below, renders higher sparsity than when using pure ${𝒟}^{\text{c}}$ or ${𝒟}^{\text{s}}$ dictionaries of the same redundancy as the combination $𝒟={𝒟}^{\text{c}}\cup {𝒟}^{\text{s}}$.

-
${𝒟}^{\text{c}}=\{w^{\text{c}}(n)\cos\frac{\pi (2i-1)(n-1)}{2M},i=1,\ldots ,L{\}}_{n=1}^{M}.$

-
${𝒟}^{\text{s}}=\{w^{\text{s}}(n)\sin\frac{\pi (2i-1)(n)}{2M},i=1,\ldots ,L{\}}_{n=1}^{M},$

where $w^{\text{c}}(n)$ and $w^{\text{s}}(n),\;n=1,\ldots ,M$ are normalization factors.

The signal representation is realized by independent approximation of each frame. The atoms ${𝐝}_{\ell_{n}^{i}},\;n=1\ldots ,k_{i}$ are selected for simultaneously representing both channels ${𝐟}_{i}\{1\}\in {\mathbb{R}}^{L}$ and ${𝐟}_{i}\{2\}\in {\mathbb{R}}^{L}$ in the *i*th frame, for i=1,…,I, with *I* the integral part of *N*/*L*. In the numerical simulations the number of atoms in each dictionary ${𝒟}^{\text{c}}$ and ${𝒟}^{\text{s}}$ is 2*L* so that the total number of atoms in $𝒟={𝒟}^{\text{c}}\cup {𝒟}^{\text{s}}$ is 4*L*.

As a metric of approximation quality we use the standard Signal to Noise Ratio (SNR) calculate as


$$SNR=10\log_{10}\left(\frac{\sum_{q=1}^{2}\frac{1}{2}\|𝐟\{q\}{\|}^{2}}{\sum_{q=1}^{2}\frac{1}{2}\|𝐟\{q\}-{𝐟}^{\text{a}}\{q\}{\|}^{2}}\right),$$

where ${𝐟}^{\text{a}}\{q\}\in {\mathbb{R}}^{N},\;q=1,2$ are the approximations of the channels $𝐟\{q\}\in {\mathbb{R}}^{N},\;q=1,2$. Since the frames are disjoint the approximation of each channel ${𝐟}^{\text{a}}\{q\}$ is obtained by the concatenation of the approximations ${𝐟}_{i}\{q{\}}^{k_{i}}$ of the corresponding frames i.e., ${𝐟}^{\text{a}}\{q\}={\hat{\text{J}}}_{i=1}^{I}{𝐟}_{i}\{q{\}}^{k_{i}},\;q=1,2$, where $\hat{\text{J}}$ indicates the concatenation operation. The numbers $k_{i},\;i=1,\ldots ,I$ of atoms for approximating each *i*-frame are decided to meet the condition:

$$\sum_{q=1}^{2}\frac{1}{2}\|{𝐟}_{i}\{q\}-{𝐟}_{i}\{q{\}}^{k_{i}}{\|}^{2}<\rho_{i}\;i=1,\ldots ,I.$$ (15)

On defining


$${snr}_{i}=10\log_{10}\left(\frac{\sum_{q=1}^{2}\frac{1}{2}\|{𝐟}_{i}\{q\}{\|}^{2}}{\sum_{q=1}^{2}\frac{1}{2}\|{𝐟}_{i}\{q\}-{𝐟}_{i}\{q{\}}^{k_{i}}{\|}^{2}}\right),\;i=1,\ldots ,I$$

the parameters $\rho_{i},\;i=1,\ldots ,I$ in (15) are determined as


$$\rho_{i}=10^{\left(-\frac{{snr}_{0}}{10}\right)}\sum_{q=1}^{2}\frac{1}{2}\|{𝐟}_{i}\{q\}{\|}^{2},\;i=1,\ldots ,I,$$

where ${snr}_{0}$ is fixed to the same value for each frame in oder to achieve the expected approximation quality.

The metric of sparsity is considered to account for the number of elements in the approximation of the whole signals. For this we define the Sparsity Ratio (SR) as


$$\text{SR}=\frac{2N}{K},$$

where $K=\sum_{i=1}^{I}k_{i}$ is the total number of atoms in the signal representation and *N* the number of samples in each of the channels. Thus, a large value of SR indicates a high level of sparsity.

The numerical example is realized using four stereo clips of melodic music: 1. Classic Orchestra. 2. Classic Guitar. 3. Chopin Piano. 4. Piazzolla Tango. All four clips are of the same length, *N* = 256000 samples in each channel (5.8 secs). The approximation is carried out on frames of length *L* = 1024 and for qualities corresponding to SNR=20 dB, SNR=25 dB, and SNR=30 dB. The improvements in the values of SR obtained with SOOMP, with respect to those obtained with SOMP, are noticeable from the comparison of the 3rd and 4th columns in Table 1 for all four clips and all approximation qualities. The 5th column displays the gain over SOMP yielded by SOOMP. The 6th and 8th columns show the approximation times (the values are the average of five independent runs with MATLAB in a Laptop Core i7-1165G7). The 7th and 9th columns give the corresponding standard deviations.

**Table 1:** Comparison of sparsity (SR values) for approximations of four clips of music up to the same SNR (20, 25 and 30 dB). The 5th column gives the gain in SR achieved by SOOMP (2) over SOMP (1). The 6th and 8th columns give the approximation times in secs. (average of five independent runs). The 7th and 9th columns are the corresponding standard deviations.

Clip	SNR	SOMP(1)	SOOMP(2)	Gain	Time (1)	std	Time (2)	std
1.	20 dB	43.3	48.4	11.8%	41.64 s	0.08	37.25 s	0.07
2.	20 dB	119.4	135.7	13.6%	15.67 s	0.11	13.88 s	0.07
3.	20 dB	67.3	74.5	10.7%	26.32 s	0.38	23.91 s	0.22
4.	20 dB	94.9	106.9	12.6%	18.42 s	0.08	16.41 s	0.02
1.	25 dB	28.0	31.3	11.8%	66.81 s	0.06	59.61 s	0.05
2.	25 dB	78.8	90.7	15.1%	24.13 s	0.13	20.93 s	0.23
3.	25 dB	45.7	51.3	12.2%	39.19 s	0.09	35.20 s	0.29
4.	25 dB	69.3	79.0	14.0%	25.53 s	0.04	22.34 s	0.03
1.	30 dB	17.9	19.8	10.6%	113.01 s	0.21	102.17 s	0.22
2.	30 dB	53.9	61.8	14.7%	35.79 s	0.27	31.02 s	0.08
3.	30 dB	32.3	36.3	12.4%	57.01 s	0.07	50.91 s	0.32
4.	30 dB	54.4	62.1	14.5%	33.06 s	0.29	28.73 s	0.06

**Fig 1:**
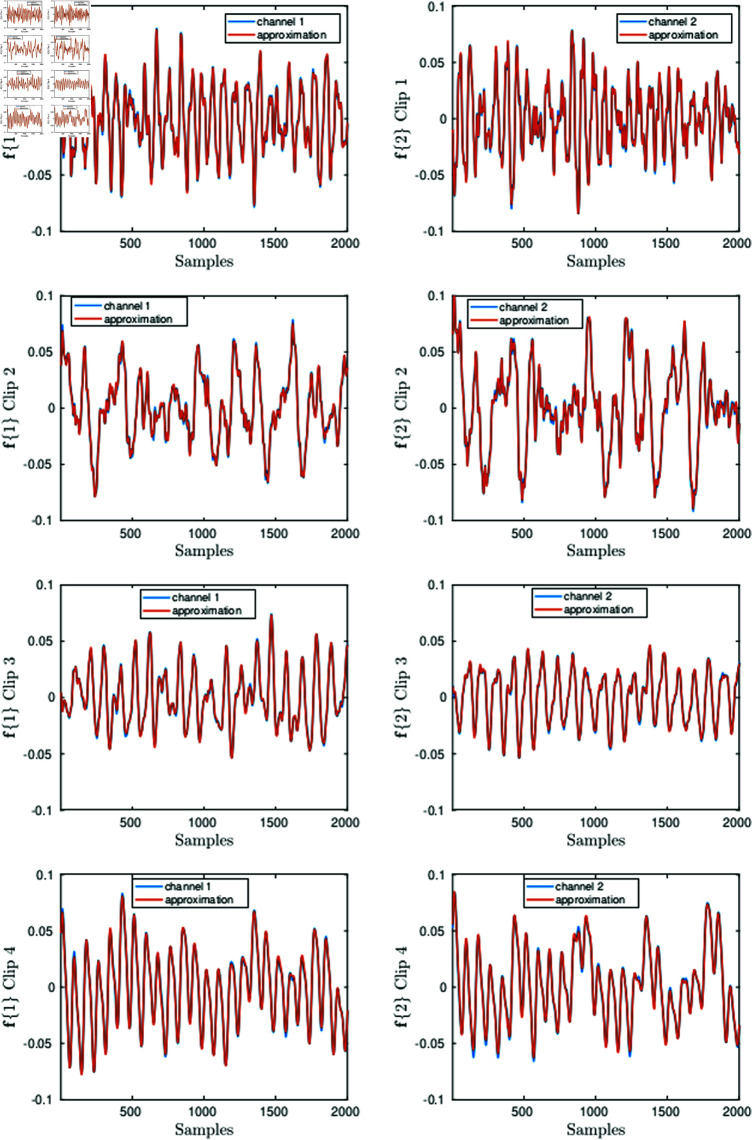
2000 samples in the Chips 1–4 (blue lines) and the corresponding approximations (red lines) up to SNR = 25 dB. The graphs on the left correspond to one of the channels and the graphs on the right to the other.

The left graphs in Fig 1 show 2000 samples in channel 1 of the original chips as well as the corresponding approximation up to 25 dB. The right graphs have the same description but correspond to the 2000 samples in channel 2. As illustrated by the graphs in Fig 1, SNR = 25 dB produces already very good pointwise approximation of the signals.

## 5 Application to compression of ECG records

**Fig 2:**
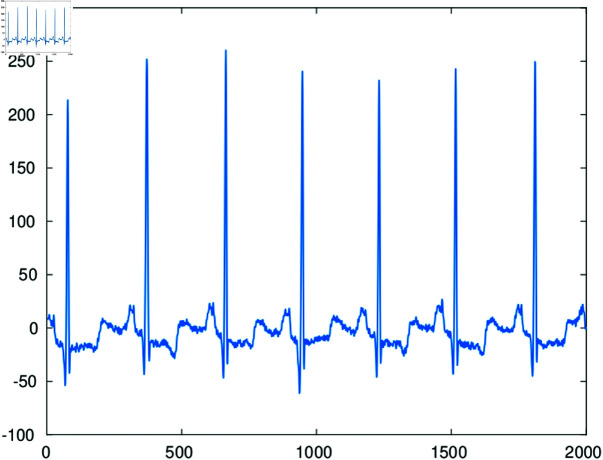
A short segment of an ECG record.

A digital ECG signal represents a sequence of heartbeats. In a typical record each heartbeat is characterized by a combination of three graphical deflections, known as QRS complex, and two lateral and less visually noticeable P and T waves. A short segment of a typical ECG record is illustrated in Fig 2.

In order to simultaneously approximate all the beats in a record we need to segment and align the beats to meet the requirement of being similar. The procedure is discussed in the next subsection.

### 5.1. Segmentation and alignment of heartbeats

**Fig 3:**
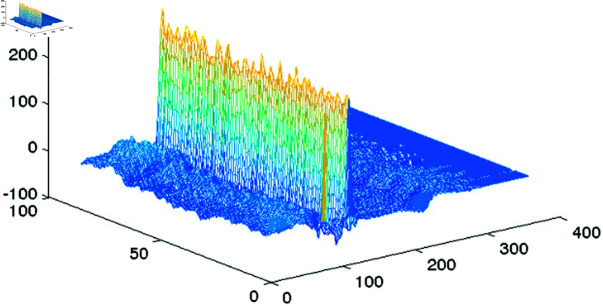
Configuration resulting by segmentation and alignment of 80 heartbeats for illustration purposes.

**Fig 4:**
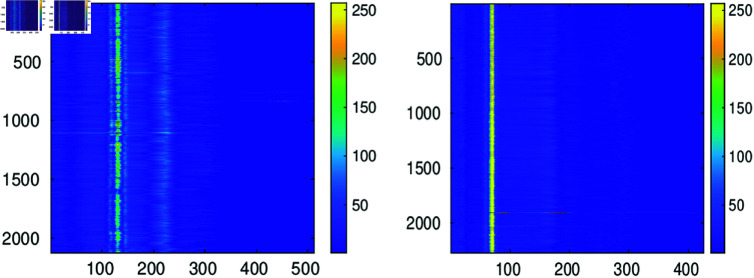
Images of the magnitude of the aligned heartbeats in records 111 (left graph) and 100 (right graph).

The QRS complex is segmented once the central R peak is detected. This can be effectively done by the Pan Tompkins method [19]. In our numerical examples we use the off-the-shelf MATLAB implementation of this algorithm [20]. Since the distance between peaks in a record is not uniform, the length of the segmented beats should be passed to the decoder. The segmented peaks are placed in arrays 𝐟{q},q=1,…,Q of equal length *L* by padding with zeros. Fig 3 illustrates the resulting configuration with 80 heartbeats. Fig 4 shows the two dimensional image of the segmented and aligned heartbeats corresponding to records 111 and 100 in the MIT-BIH Arrhythmia database [21].

For simultaneously approximating heartbeats we use a wavelet dictionary. Given a partition $x_{i},\;i=1,\ldots ,N$ of the interval [*c*, *d*] the dictionary is constructed as follows [22,23].

$$𝒟={𝒱}_{0}\cup {𝒲}_{0}\cup {𝒲}_{1}\cup {𝒲}_{2}\cup {𝒲}_{3}\cup {𝒲}_{4},$$ (16)

with

$${𝒱}_{0}=\{\phi (x_{i}-\frac{k}{2}){|}_{\left[c,d\right]},\;k\in \mathbb{Z},\;i=1,\ldots ,N\},$$ (17)

and

$${𝒲}_{j}=\{2^{j/2}\psi (2^{j}x_{i}-\frac{k}{2}){|}_{\left[c,d\right]},\;k\in \mathbb{Z},\;i=1,\ldots ,N\},$$ (18)

where $\psi (2^{j}x_{i}-\frac{k}{2}){|}_{\left[c,d\right]}$ indicates the restriction of the function $\psi (2^{j}x_{i}-\frac{k}{2})$ to the interval [*c*, *d*]. Different families of wavelet basis and dictionaries for approximation of heartbeats have been compared in [22], where the Cohen–Daubechies–Feauveau family was singled out as the most effective one. We have confirmed the same outcome for simultaneous approximations and adopted the Cohen–Daubechies–Feauveau CDF97 dictionary of redundancy approximately two introduced in [22].

**Fig 5:**
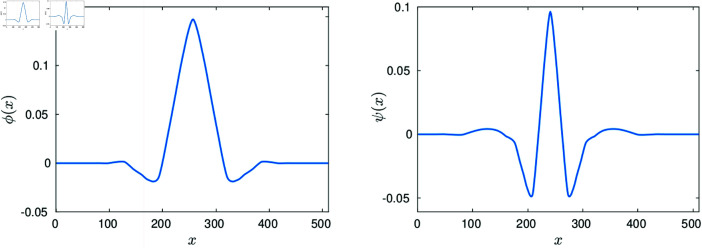
Cohen–Daubechies–Feauveau scaling and wavelet functions [24].

The prototype functions ϕ(x) and ψ(x) are plotted in the left and right graphs of Fig 5 respectively. The MATLAB codes for producing numerically both functions and building the dictionary (16) are described in [23]. The codes have been made available in [25] together with of the complete MATLAB software for reproducing the numerical examples in this work.

The segmented and aligned heartbeats are simultaneously approximated using the SOOMP approach by assigning the same weight to each heartbeat, i.e. $p(q)=\frac{1}{Q},\;q=1,\ldots ,Q$. In this case the algorithm stops at iteration *k* if


$$\sum_{q=1}^{Q}\frac{1}{Q}\|𝐟\left\{q\right\}-𝐟{\left\{q\right\}}^{k}\|<\rho ,$$

with

$$\rho =\frac{{PRDN}_{0}}{100}\sum_{q=1}^{Q}\frac{1}{Q}\|𝐟\left\{q\right\}-\overset{―}{𝐟\left\{q\right\}}\|,$$ (19)

$\overset{―}{𝐟\left\{q\right\}}$ indicating the mean of 𝐟{q} and ${PRDN}_{0}$ a fixed value of the metric of quality PRDN, which is defined by

$$PRDN=\frac{\left\|𝐟-{𝐟}^{a}\right\|}{\left\|𝐟-\overset{―}{𝐟}\right\|}\times 100\%,$$ (20)

where 𝐟 is the whole ECG record, ${𝐟}^{a}$ is the reconstructed record from the approximated heartbeats and $\overset{―}{𝐟}$ is the mean of 𝐟.

Given a required value of PRDN, at the approximation step of the processing the parameter ${PRDN}_{0}$ is fixed as 0.8 · PRDN, in order to achieve the target value PRDN at the quantization step described in Subsect 5.2.

**Fig 6:**
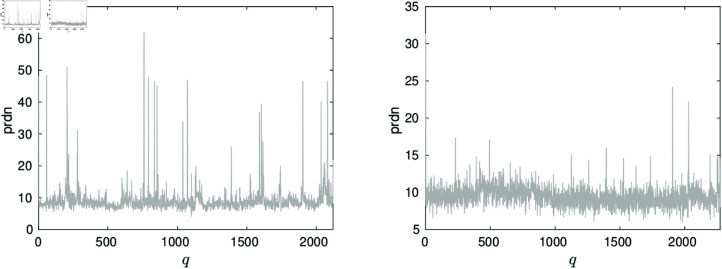
Values of prdn from the simultaneous approximation of the aligned beats in records 111 (left graph) and 100 (right graph).

Even if the approximation is realized to achieve the required ${PRDN}_{0}$ by the whole record, it is interesting to calculate the quality metric for each heartbeat in the array. To this end we defined

$$prdn(q)=\frac{\left\|𝐟\left\{q\right\}-{𝐟}^{a}\{q\}\right\|}{\left\|𝐟\left\{q\right\}-\overset{―}{𝐟\left\{q\right\}}\right\|}\times 100\%,\;q=1,\ldots ,Q,$$ (21)

where ${𝐟}^{a}\{q\}$ is the approximation of the beat 𝐟{q} and $\overset{―}{𝐟\left\{q\right\}}$ its mean value. The values of prdn(q) for the simultaneous approximation of records 111 and 100 are shown in the left and right graphs of Fig 6 respectively.

The total PRDN produced by the reconstruction of the whole records is, in both cases, PRDN=9.1. The mean value prdn for record 111 is 9.0 with std=4 while for record 100 the mean prdn is 9.4 with std=1.4. However, as noticeable in the figures, for some *q*s the prdn values are much higher than for others. This is a consequence of the irregularities of the beats, which can be perceived in the left image of Fig 4.

**Fig 7:**
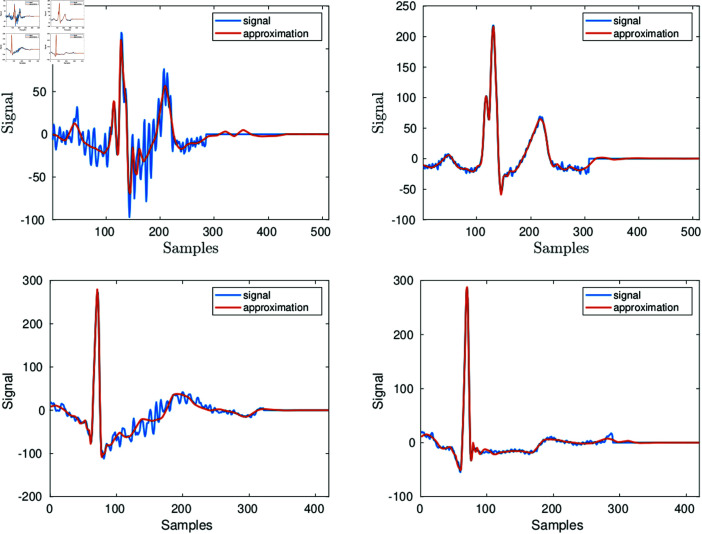
The top left graph shows one of the heartbeats in records 111, and its approximation, which yields an outlier value of prdn. The right graph corresponds to a beat yielding prdn close to the mean value prdn=9. The bottom graphs have the same description but the heartbeats are from record 100.

The top left graph of Fig 7 depicts a heartbeat in records 111 and its approximation. This heartbeat yields prdn=46.5. As shown by the red line the figure, such high prdn value is produced by a smooth version of the noisy signal. On the contrary, for regular heartbeats the prdn values are close to the PRDN of the whole record. The right graph of the figure shows the approximation of one of those beats. The left bottom graph is the approximation of one of the few beats in record 100 which yields the outlier value prdn=20. The right bottom graph shows the approximation of one of the other beats.

**Fig 8:**
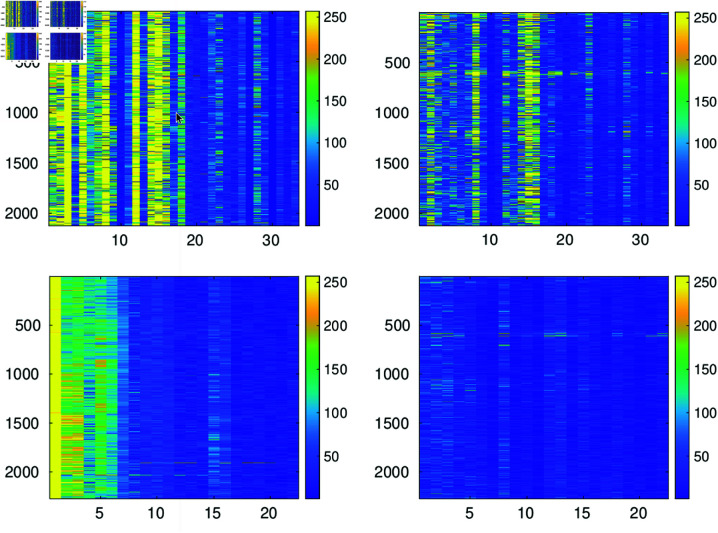
The top image on the left depicts the magnitude of entries in the array $𝐂\in {\mathbb{R}}^{2133\times 33}$ containing the coefficients in the approximation of record 111. The bottom left image depicts the magnitude of entries in the arrays $𝐂\in {\mathbb{R}}^{2273\times 25}$ corresponding to the approximation of records 100. The images on the right are the magnitude of the entries in the arrays $𝐁\in {\mathbb{R}}^{2133\times 33}$ (top) and $𝐁\in {\mathbb{R}}^{2273\times 25}$ (bottom) arising by applying the discrete cosine transform on the columns of the arrays represented by the images on the left.

By the simultaneous approximation of the aligned heartbeats these are transformed into a reduced set of numbers which allow to reconstruct the approximated heartbeats. This set consists of a) the *k* indices $\ell_{n},\;n=1,\ldots ,k$ corresponding to the common atoms in the decomposition of the heartbeats (c.f. (1)) b) the different coefficients c{q}(n),n=1,…,k,q=1,…,Q in the decomposition of each heartbeat (c.f. (1)). These coefficients can be placed in a two dimensional array $𝐂\in {\mathbb{R}}^{Q\times k}$ as illustrated in Fig 8. The top image on the left in this figure shows the magnitude of the array $𝐂\in {\mathbb{R}}^{2133\times 33}$ arising from the approximation up to PRDN=9 of record. 111. The bottom left image corresponds to the approximation up to PRDN=9 of record 100.

It is clear from the location of the brightest pixels in the left images of Fig 8 that the coefficients of largest magnitude are concentrated in vertical lines. This suggests that, to favor compression for storing these values, it is convenient to apply an orthogonal transformation to map the coefficients in the vertical direction to smaller values which eventually might be quantized to zero. Consequently, by applying the discrete cosine transform on each column of 𝐂, we create the transformed array $𝐁\in {\mathbb{R}}^{Q\times k}$ with the following entries

$$𝐁(:,n)=\hat{dct}𝐂(:,n),\;n=1,\ldots ,k,$$ (22)

where $\hat{dct}𝐂(:,n)$ indicates the one dimensional discrete cosine transform operating on the *n*-th column of array 𝐂. The transformed points corresponding to the left images in Fig 8 are represented in the right images of this figure. The introduction of this step to decorrelate the vertical entries in the array 𝐂 is key to boost the performance of the adopted encoding strategy described below. The notorious change of intensity in the images on the right of Fig 8 indicates that after quantization some of the entries of the transformed arrays will be mapped to zero. Within the encoding strategy described in the next section, this effect enhances compression.

### 5.2. Encoding

At the encoding step the Q×k array 𝐁 is expressed as a vector 𝐛=(b(1),…,b(K)) of K=Q·k components, adopting the column-major order. The encoding of this vector follows the procedure outline in [26]. The components of 𝐛 are converted to integer numbers by a mid-tread uniform quantizer as follows:

$$b^{\Delta }(i)=\left\lfloor \frac{b(i)}{\Delta }+\frac{1}{2}\right\rfloor ,\;i=1,\ldots ,K,$$ (23)

where ⌊x⌋ indicates the largest integer smaller or equal to *x* and Δ is the quantization parameter. For comparison with results in other publications in the numerical examples the quantization parameter Δ is set to produce the required quality of the reconstructed signal.

The absolute value of the elements (23) are placed in a smaller vector, say $b^{\prime }=(b^{\prime }(1),\ldots ,$ $b^{\prime }(K^{\prime }))$, after the elimination of zeros. The signs are encoded separately in a vector $𝐬=(s(1),\ldots ,s(K^{\prime }))$ using a binary alphabet: 1 for + and 0 for –.

Assuming that the nonzero values in (23) occur at the positions $j_{i},\ldots ,j_{K^{\prime }}$, these indices are re-ordered in ascending order $j_{i}\to {\tilde{j}}_{i},\;i=1,\ldots ,K^{\prime }$, i.e. ${\tilde{j}}_{i}<{\tilde{j}}_{i+1},\;i=1,\ldots ,K^{\prime }$. This induces new order in the coefficients, $b^{\prime }\to {\tilde{b}}^{\prime }$ and in the corresponding signs $𝐬\to \tilde{s}$. Defining $\delta (i)={\tilde{j}}_{i}$ − ${\tilde{j}}_{i-1},\;i=2,\ldots ,K^{\prime }$ the array $\delta =({\tilde{j}}_{1},\delta (2),\ldots ,\delta (K^{\prime }))$ stores the indices ${\tilde{j}}_{1},\ldots ,{\tilde{j}}_{K^{\prime }}$ with unique recovery.

Finally the vectors ${\tilde{b}}^{\prime },\tilde{s},\delta$, as well as the length of the heartbeats 𝐡, are compressed using adaptive Huffman coding implemented by the off-the-shelf MATLAB function Huff06 [27]. The additional numbers which have to be passed to the decoder are:

(i) The indices $\ell_{i},\;i=1,\ldots ,k$ of the selected dictionary’s atoms forming the common basis.
(ii) The quantization parameter Δ.
(iii) The mean value of the 1D ECG record (if not previously subtracted).
(iv) The number of rows and columns of 𝐂, i.e. *Q* and *k*.

### 5.3. 1D ECG signal recovery

At the decoding stage, after reverting Huffman coding, the locations ${\tilde{j}}_{1},\ldots ,{\tilde{j}}_{K}$ of the nonzero entries in the transformed array after quantization are readily obtained. This allows the recovery of the array ${𝐁}^{r}$ as follows.

(i) Set $b^{r}(i)=0,\;i=1,\ldots ,K$ and $b^{r}({\tilde{j}}_{i})=(2\tilde{s}(i)-1){\tilde{b}}^{\prime }(i)\Delta ,\;i=1,\ldots ,K$.
(ii) Reshape the vector ${𝐛}^{r}$ to produce a 2D array ${𝐁}^{r}$ of size Q×k. The array ${𝐂}^{r}$ is recovered from the ${𝐁}^{r}$ one by inverting the $\hat{dct}$ transformation (c.f. (22)).
(iii) Each row of the recovered array ${𝐂}^{r}$ gives the coefficients in the decomposition (1) of the approximated heartbeats, i.e. ${𝐟}^{\text{r}}\{q\}=\sum_{i=1}^{k}{𝐂}^{r}(q,i){𝐝}_{\ell_{i}},\;q=1,\ldots ,Q.$
(iv) Finally the reconstructed beats ${𝐟}^{\text{r}}\{q\}$ are assembled in a 1D record using the distance between heartbeats that was stored in the vector 𝐡.

The achieved compression ratio CR, which is defined as

$$\text{CR}=\frac{\text{Size of the uncompressed file}}{\text{Size of the compressed file}},$$ (24)

depends on the required quality of the recovered signal. In the numerical examples the quality of the recovered records is assessed by the PRDN as defined in (20). It is pertinent to stress the importance of adopting this normalized metric for comparison of reconstruction quality. The subtraction of $\overset{―}{𝐟}$ avoids dependence on the signal baseline.

## 6 Numerical tests

For the numerical test we use the MIT-BIH Arrhythmia database [21]. Each of the records is of 30 min length, consisting of *N* = 650000 11-bit samples at a frequency of 360 Hz.

For comparison purposes we compress the subset of records reported in [28,29], and [30] and reproduce the values of PRDN in those publications. This is achieved as follows: The SOOMP method is applied to approximate the set of heartbeats in each record up to 80% the target PRDN. The quantization parameter Δ is automatically fixed, by a bisection algorithm, in order to reproduce the target PRDN for the whole record within two decimal places.

The first, second and third columns of Table 2 reproduce the results published in [28]. The comparison is relevant because the approach [28] is also based on approximation of heartbeats using a dictionary. The techniques are very different though. Whilst our dictionary does not have to be stored because it is numerically generated, the dictionary in [28] is part of the ECG record to be compressed. Moreover, the method for finding the sparse representation is different and so is the procedure to store the parameters that should be passed to the decoder.

**Table 2:** Comparison with the results in [28]. The first collumn lists the records considered in [28]. The second column displays the values of PRDN and the third collum their CRs. Our CRs for the same PRDN are shown in the forth column. The fifth column shows the CRs obtained with the fast approach [26].

Record	PRDN	CR [28]	CR prop.	CR [26]
100	18.03	78.20	143.99	36.51
100	17.22	75.12	139.47	35.25
101	14.66	80.24	102.58	31.26
101	12.91	76.46	82.31	30.31
102	18.54	58.54	58.49	33.89
102	18.16	48.47	58.13	33.48
103	12.57	46.32	90.91	30.84
103	11.57	44.33	86.27	29.61
109	13.70	24.86	145.80	51.23
109	9.97	23.53	97.73	36.91
111	26.20	31.05	121.09	38.29
111	19.51	29.44	60.49	32.20
112	16.58	34.06	91.48	35.05
112	15.99	35.49	85.59	34.32
113	14.08	37.42	90.76	32.49
113	9.82	32.55	55.30	27.68
115	9.76	38.26	62.31	24.52
115	9.18	36.57	57.32	23.74
117	14.42	38.94	120.89	36.94
117	13.38	37.13	105.97	35.74
119	32.19	16.26	153.33	90.40
119	16.36	15.24	78.81	48.08
121	17.36	26.67	111.74	46.45
121	15.63	25.29	100.72	41.11
Average	17.33	41.9	107.78	40.65
Average	14.14	39.97	84.00	34.04

Our compression results are shown in the forth column of Table 2. These results demonstrate a significant gain in CR for the same recovery quality. For further comparison we apply the fast compression algorithm [26], which does not require peak segmentation or Huffman coding. This method has been already shown to improve the average CR for the 48 records in the MIT-BIH Arrhythmia dataset with respect to the results in [31,32], and [33], for a broad rage of average qualities. For comparison with [28] in Table 2 the compression is realized to reproduce the PRDN listed in the second column for each record.

The first, second and third columns of Table 3 reproduce the results published in [29], which are achieved with an approach based on the Singular Value Decomposition (SVD).

**Table 3:** Same description as in Table 2 but the comparison is with the results of Table I in [29].

Record	PRDN	CR [29]	CR prop.	CR [26]
100	11.46	39.81	64.47	23.03
101	14.13	42.04	95.53	30.91
102	19.94	41.09	63.69	35.09
103	6.72	41.24	39.17	21.05
107	13.27	41.84	71.40	38.15
109	7.31	38.25	59.34	28.46
111	13.94	41.73	41.59	25.14
115	8.04	42.71	47.79	22.04
117	10.00	46.75	51.56	26.17
118	15.33	39.60	66.44	28.26
119	9.67	41.97	43.98	30.71
213	13.63	32.58	62.24	25.72
222	22.44	40.69	31.95	24.77
232	20.76	42.28	59.66	35.52
Average	13.33	40.90	57.09	28.22

Our compression ratios (CRs) are shown in the the forth column of Table 3. The fifth column shows the CRs produced by the fast compression algorithm [26].

The first, second and third columns of Table 4 reproduce the results published in [30], which are also obtained with a Singular Value Decomposition based approach. Our CRs are shown in the forth column of this table. The fifth column shows the CRs produced by the fast compression algorithm [26].

**Table 4:** Same description as in Table 2 but the comparison is with the results of Table I in [30].

Record	PRDN	CR [30]	CR prop.	CR [26]
100	11.55	50.70	65.47	23.14
101	11.29	58.54	64.13	27.86
103	9.16	54.16	61.37	25.70
107	14.53	53.12	78.31	40.34
109	11.83	46.43	120.08	44.67
111	16.40	53.39	50.11	28.34
115	8.94	56.77	58.76	23.49
117	12.43	66.15	90.39	34.62
119	10.28	56.03	46.99	32.17
214	17.03	50.84	87.66	54.04
223	17.49	45.38	86.06	40.80
Average	12.81	53.77	73.57	34.11

Note: The MATLAB software for reproducing the tables is available on http://www.nonlinear-approx.info/examples/node017.html

## 7 Conclusions

The Optimized Orthogonal Matching Pursuit approach has been extended with the purpose of selecting a common basis for the simultaneous approximation of a set of similar signals. The extended approach, termed Simultaneous Optimized Orthogonal Matching Pursuit, minimizes at each iteration the mean value square error norm of the joint approximation. The algorithm’s implementation was demonstrated by approximating stereophonic music using a highly coherent trigonometric dictionary. The applicability of the method to ECG compression was illustrated on records taken from the MIT-BIH Arrhythmia database. The particular records were selected for comparison purposes as in [28,29], and [30]. The simultaneous approximations of aligned heartbeats was used for compressing a whole record. The adopted compression strategy was shown to improve upon compression results achieved by other methods for the same reconstruction quality. The comparison was made possible by means of an iterative quantization procedure which delivers the required quality.

While the proposed approach involves detection and alignment of R-peaks, it is the approximation step which introduces the highest computational cost. In order to address this matter as a line of future work, it would be interesting to investigate the possibility of selecting a suitable subspace $V_{k}$ from a whole data set (instead of a subspace for each single record as is done here). Certainly approximating new records using previously selected atoms would significantly speed the compression procedure. It is still to be discerned if the SOOMP approach could pick out a common low dimension subspace to approximate, up to a given quality, any new ECG record. We feel confident that the results presented in this work will motivate further research in the topic.
